# Opportunities and challenges of proximity labeling for microbe-host cell interactions in tumor microenvironment

**DOI:** 10.3389/fcimb.2025.1723709

**Published:** 2026-01-26

**Authors:** Shuang Qiu, Kaihong Wang, Amin Sun, Haifu Sun, Xiang Li, Chun Xia Chen

**Affiliations:** 1 Key Laboratory of Forest Plant Ecology, Northeast Forestry University, Ministry of Education, Harbin, Heilongjiang, China; 2 College of Chemistry, Chemical Engineering and Resource Utilization, Northeast Forestry University, Harbin, Heilongjiang, China; 3 Hubei Province Key Laboratory of Biotechnology of Chinese Traditional Medicine, College of Health Science and Engineering, Hubei University, Wuhan, China

**Keywords:** microbe-host cell interaction, proximity labeling, enzyme and photocatalyst, interaction mechanism, drug screening

## Abstract

In the tumor immune microenvironment, microbes promote tumor progression and metastasis by invading host cancer cells. Blocking these interactions is expected to provide new strategies for inhibiting tumor progression and metastasis, as well as opening up new avenues for immunotherapy. However, technological means of studying the interaction between microorganisms and host cancer cells are still limited. Proximity labeling, a widely used method for analyzing biomolecular and cellular interactions, has the potential to analyze microbe-host cell interactions quantitatively, uncovering the key factors that influence these interactions within the tumor immune microenvironment in order to control tumor initiation and progression. Furthermore, proximity labeling based strategies can be applied to high-throughput drug screening aimed at disrupting pathogenic microbe-host interactions, contributing to the development of therapeutics against advanced and metastatic tumors. This paper provides a systematic review of the topic, introducing cutting-edge microbiological mechanisms that have attracted the attention of oncologists.

## Introduction

Tumor immunotherapy is a new generation of tumor therapies developed after traditional cancer treatments such as surgery, radiotherapy and chemotherapy. The main methods for tumor immunotherapies include immune checkpoint inhibitor therapy, T-cell therapy and tumor vaccines (Zhang and Zhang, 2020), providing great potential for application in clinical cancer treatment. In the process of tumor immunotherapy, understanding the interactions between different cells and their mechanisms in the immune microenvironment is crucial for tumor treatment (Waldman et al., 2020). Initially, it was widely believed that tumor tissue was a sterile environment, but clinical studies have confirmed that microorganisms are present in tumor tissue and these microorganisms have a variety of effects on tumor biology during tumorigenesis, metastasis and treatment (Sepich-Poore et al., 2021). In addition to tumor cells and important immune cells such as T cells, antigen-presenting cells, phagocytes and neutrophils, resident microorganisms are also an important part of the tumor microenvironment. These microorganisms are widely present in different types of cancer, such as pancreatic cancer (Pushalkar et al., 2018), lung cancer (Jin et al., 2019), breast cancer (Fu et al., 2022), hepatocellular cancer (Sun et al., 2023), etc., and interact with host cells in the microenvironment. Microbe-host interactions influence tumor initiation, progression and the patient’s response to chemotherapy or immunotherapy.

## The role of microbes in tumor progression and metastasis

The study of complex interactions between microbes and host cells has expanded from symbiosis in most human tissues to cancer development, a process closely related to human malignancies (Wu et al., 2025). The presence of commensal bacteria directly affects tumor initiation, progression and therapeutic responses. The symbiotic flora in endocrine tissues is an important component of the tumor immune microenvironment and may have a more significant impact on tumor initiation and progression at a local level (Zheng et al., 2020).

In 2020, researchers from the Weizmann Institute confirmed the presence of bacteria within tumors, mainly between cancer and immune cells, and found that different tumor samples contain different types of bacteria (Nejman et al., 2020). In 2022, Cai et al. reported the specific “intracellular bacteria” in breast cancer tissues for the first time, which play a role in tumor colonization and metastasis (Fu et al., 2022).

**Figure 1:**
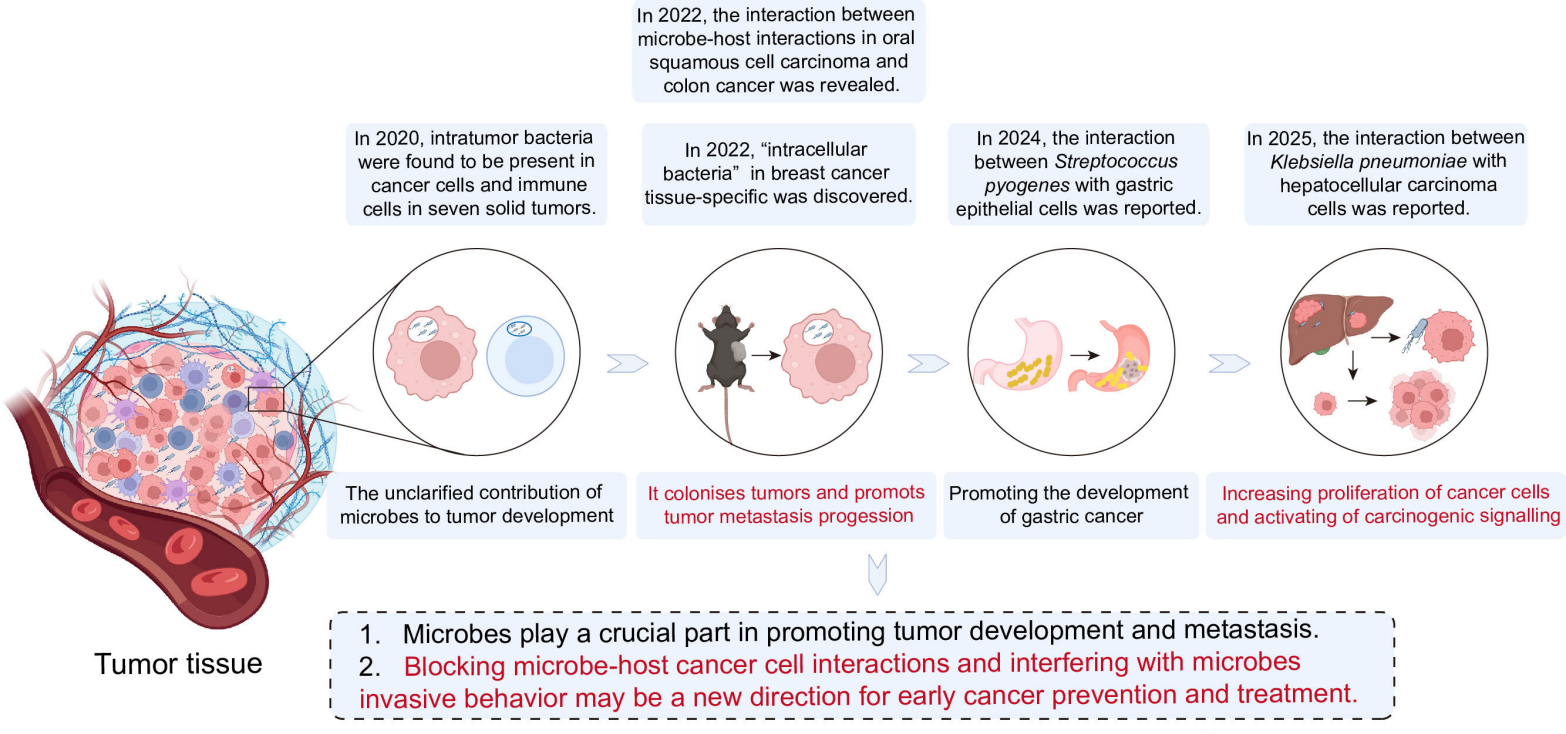
Advances in the study of the role of microbes in promoting tumor development and metastasis.

In 2022, Susan Bullman et al. used single-cell sequencing tools and spatial transcriptome technologies to reveal microbe-host interactions in oral squamous cell carcinoma and colon cancer. In 2024, Yu et al. reported that *Streptococcus pyogenes*, a non*-Helicobacter pylori* pathogen, promotes gastric carcinogenesis by interacting with gastric epithelial cells (Fu et al., 2024). The following year, they also revealed further details about the interaction between *Klebsiella pneumoniae* and hepatocellular carcinoma cells, which leads to increased cancer cell proliferation (Wang et al., 2025) (**Figure 1**). Collectively, these studies collectively suggest that microbes play a pivotal role in tumor development and progression.

Microbes play a significant role in tumor progression and metastasis through a series of sophisticated molecular mechanisms. These mechanisms form a complex network that reshapes the tumor microenvironment, creating conditions that encourage the proliferation, invasion and distant metastasis of cancer cells. Certain microbes can directly induce genotoxicity and genomic instability. For example, colibactin toxins produced by certain Escherichia coli strains induce double-strand breaks in host cell DNA, disrupting cell cycle checkpoints and driving the initiation of colorectal cancer (Canzian et al., 2025). Additionally, microbes and their metabolites shape immunosuppressive microenvironments by modulating immune responses. Recent studies have also revealed that the metabolite Nα-acetyl-L-lysine, produced by Staphylococcus sciuri, can be catalyzed by lysyl oxidase homolog 2 (Loxl2) to produce hydrogen peroxide (H_2_O_2_). This low-level reactive oxygen species activates the JNK signaling pathway, triggering the DNA damage response pathway and ultimately promoting cell division in a manner similar to tumor proliferation. This mechanism has been validated in both Drosophila models and human colon cancer cell lines (Geng et al., 2025). Furthermore, microorganisms pave the way for metastasis by activating specific pro-inflammatory and pro-metastatic signaling pathways. Bacterial lipopolysaccharide (LPS) binds to Toll-like receptor 4 (TLR4) on host cells, initiating the MyD88/NF-κB signaling pathway downstream. This triggers the release of numerous pro-inflammatory factors (e.g. IL-6 and TNF-α), creating an inflammatory microenvironment that is conducive to tumor cell invasion and metastasis (Zhang and Liu, 2025).

Thus, microbes can comprehensively influence tumor progression comprehensively—from initiation and promotion to metastasis—through multiple molecular mechanisms, including direct genotoxicity, immune regulation, metabolite-mediated effects and activation of key signaling pathways.

Blocking microbe-host cell interactions to prevent host cell invasion emerges as a promising approach for the early prevention of metastasis and progression of various cancers, providing an alternative to the treatment of advanced tumors. Understanding how these microorganisms interact with host cells in the immune microenvironment prior to invasion, and how drugs can intervene in these interactions, will help researchers to regulate microbe-host cell interactions in the tumor microenvironment. This could be achieved by using drugs correctly in clinical practice to control the invasion process of tumor cells in the pre-metastatic stage of tumor development and block the further development and metastasis of cancers.

## Limitations of existing research methods

Despite the crucial role of microbe-host cell interactions in both basic scientific research and clinical therapies, convenient and usable research tools are lacking. The main tools used to study these interactions are microscopic imaging techniques, which capture processes such as the invasion of cells by bacterial pathogens (Shi et al., 2020). Other techniques include using transcriptome or spatial transcriptome sequencing to understand changes in the interaction between microorganisms and host cells (Westermann and Vogel, 2021; Lötstedt et al., 2024; Saarenpää et al., 2023); and using bioinformatics databases to predict interactions (Wang et al., 2018). These existing tools can be used to study the interaction between microorganisms and host cells. However, most of them have some limitations:

1. Microscopic imaging techniques require the sample to be fixed or dehydrated, and if the resolution is insufficient, only the existence of the interaction can be observed rather than a quantitative study of the interaction and changes.
2. Transcriptome sequencing techniques can only really be used to analyze changes in the average transcript level of microorganisms or host cells after the interaction.
3. Bioinformatic prediction tools can provide information on microbe-host cell interactions, the strength of these interactions and the reliability of their mechanisms need to be verified.

**Figure 2:**
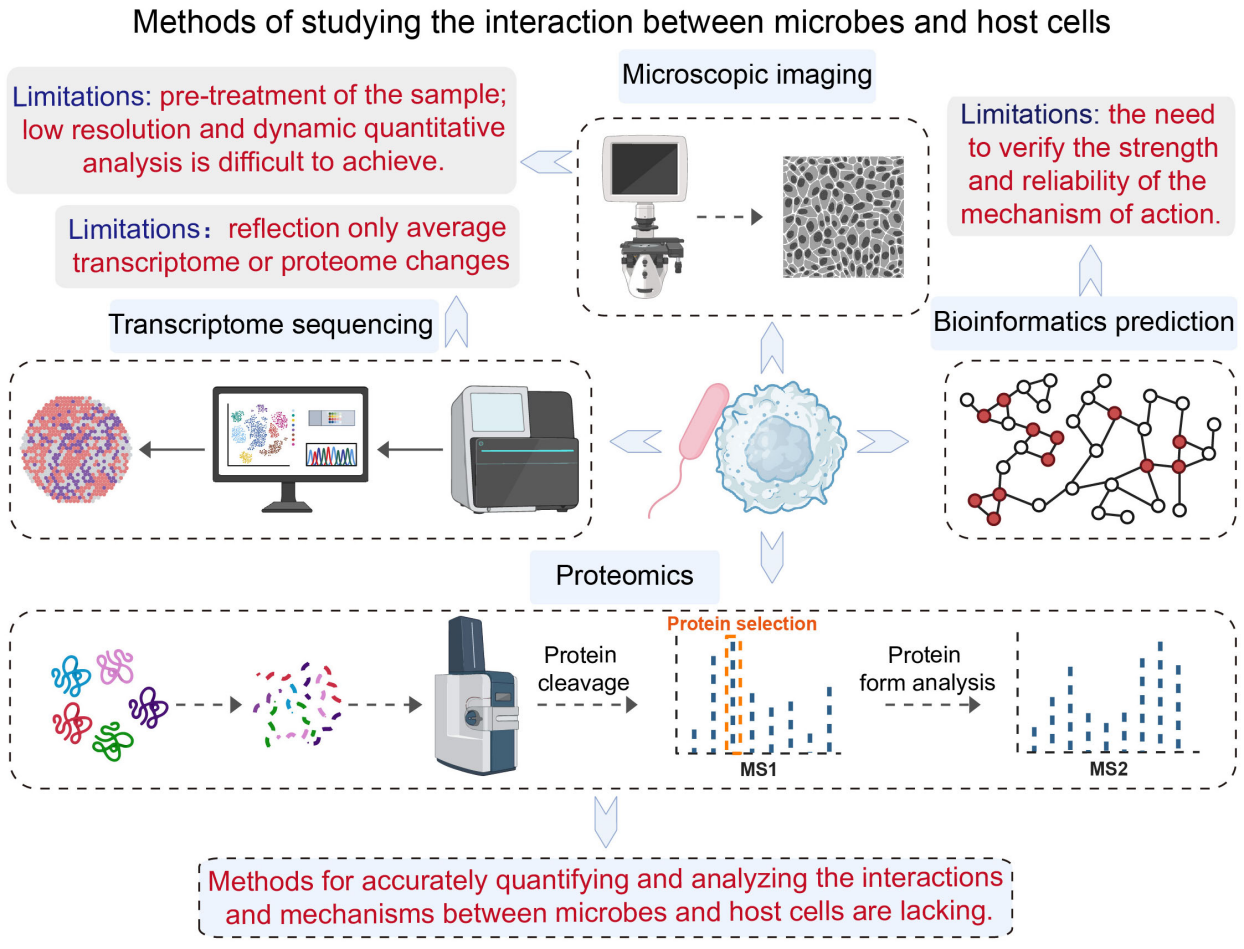
Current approaches for studying the interaction between microbes and host cells.

Clearly, there is a need for methods that can accurately quantify these interactions and elucidate their mechanisms. It is also necessary to develop quantitative research tools that can efficiently capture these interactions and analyze their mechanisms in depth (**Figure 2**).

## Advantages and application potential of proximity labeling techniques

**Figure 3:**
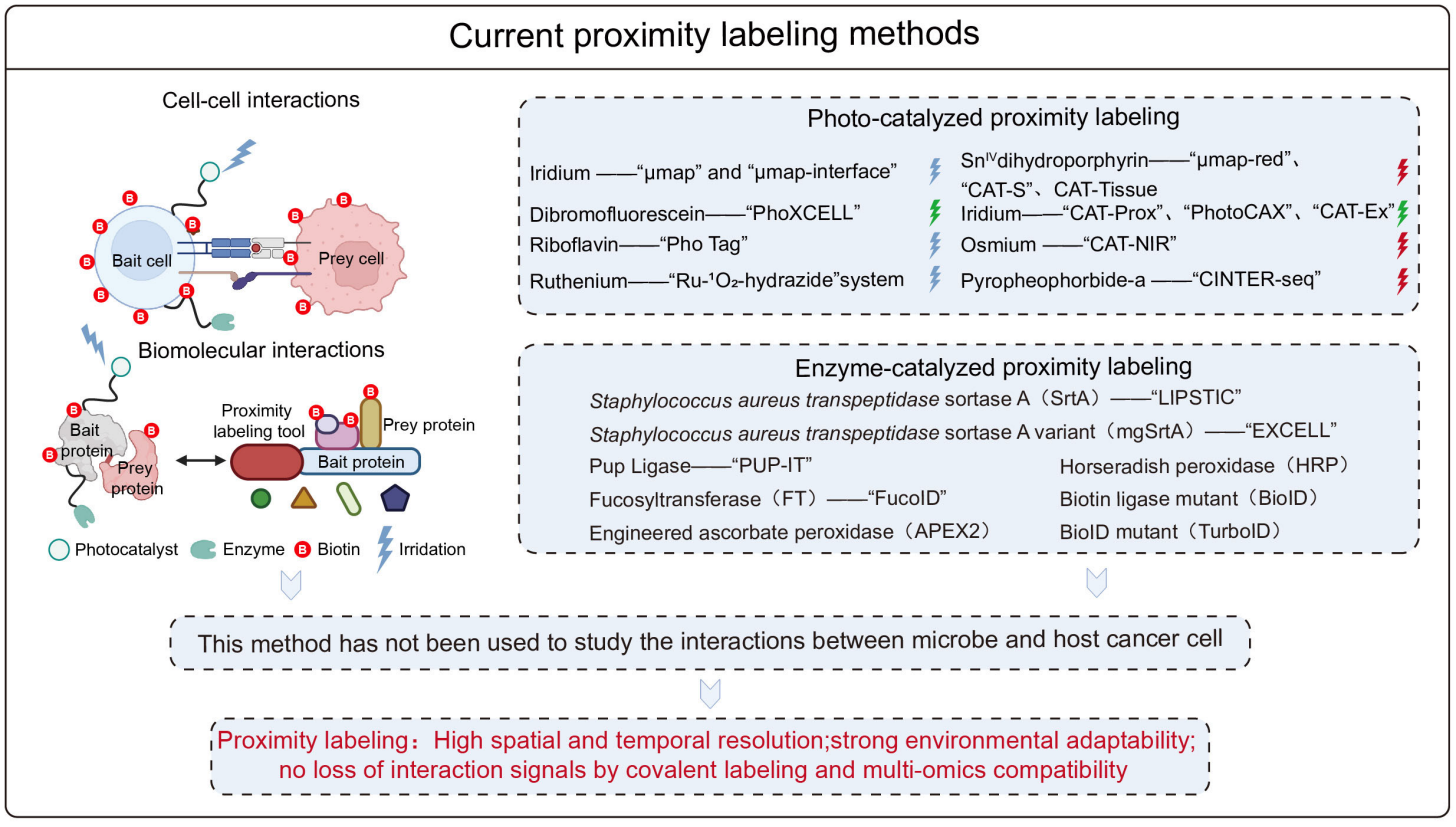
Summary of available proximity labeling tools for studying biomolecule-to-molecule and cell-cell interactions.

In recent years, proximity labeling techniques have become an important tool in chemical biology for the study of biomolecular interactions (Qin et al., 2021; Jablonska, 2024) and cell-cell interactions (Bechtel et al., 2021). There are two types of proximity labeling techniques: enzyme-catalyzed (Choi and Rhee, 2022) and photocatalyzed strategies (Fang and Zou, 2023; Knutson et al., 2024). The main enzyme-catalyzed proximity labeling techniques developed to date include the LIPSTIC (Pasqual et al., 2018; Chudnovskiy et al., 2024; Nakandakari-Higa et al., 2024) strategy based on *Staphylococcus aureus* transpeptidase sortase A (SrtA), the EXCELL (Ge et al., 2019) strategy based on a highly active mutant of the transpeptidase (mgSrtA), the PUP-IT (Liu et al., 2018) technology based on Pup ligase enzymes, and the FucoID (Liu et al., 2020; Qiu et al., 2022) technology based on fucosyltransferase for studying cell-cell interactions. The Engineered Ascorbate Peroxidase (APEX2) (Rhee et al., 2013; Loh et al., 2016; Dumrongprechachan et al., 2021; Golas and Chory, 2023; Qu et al., 2025) and Horseradish Peroxidase (HRP) (Li et al., 2020) proximity labeling techniques are used to study biomolecular interactions, as are the Biotin Ligase Mutant (BioID) (Roux et al., 2012) and BioID Ligase Mutant (TurboID) (Branon et al., 2018; Cho et al., 2020; Kwak et al., 2020; Shafraz et al., 2020) techniques. Photocatalytic proximity labeling techniques include the μmap strategy (Geri et al., 2020; Huth et al., 2023), which is based on metallic iridium catalysts, the PhoXCELL (Liu H. et al., 2022) technique, which is based on dibromofluorescein, the Pho Tag (Oslund et al., 2022) technique based on riboflavin and the “Ru- (Zhang and Zhang, 2020)O_2_-hydrazide” system (Qiu et al., 2023), which is based on metallic ruthenium complexes. It also includes the recently reported “CINTER-seq” technique with pyro pheophorbide-a (PPa) *in vivo* for capturing cell-cell interactions (Deng et al.). These techniques are used to study cell-cell interactions. The Sn^IV^ dihydroporphyrin e6 catalyst μmap-red technique (Buksh et al., 2022), metal-iridium catalyst based CAT-Prox (Huang et al., 2021), PhotoCAX (Luo et al., 2022), CAT-Ex (Liu Z. et al., 2022), CAT-S (Liu et al., 2024), dihydroporphyrin e6 catalyst based CAT-Tissue (Lou et al., 2025) and metal-osmium based NIR catalyzed bioorthogonal reactions (Liang et al., 2023) can be used to study biomolecular interactions. These methods have provided a comprehensive set of tools for studying both types of interactions. However, they have not been utilized to study microbe-host cell interactions, offering a powerful tool for investigating the interactions between the two (**Figure 3****).**

Proximity labeling techniques represent a paradigm shift in the study of microbe-host interactions, overcoming the limitations of conventional methods. Unlike microscopic imaging, which often requires sample fixation or dehydration—processes that can alter naive cellular structures and dynamics—proximity labeling operates in living cells, capturing protein interactions in their authentic physiological context. When imaging resolution is insufficient, it can only confirm colocalization, not direct molecular partnership. In contrast, proximity labeling provides a definitive nanometer-scale “snapshot” of the interacting proteome, which can be analyzed quantitatively via mass spectrometry to measure interaction dynamics, not just their existence. Furthermore, while transcriptome sequencing is invaluable, it only infers the consequences of interactions by reporting averaged changes in gene expression after the event. This blurs cell-to-cell heterogeneity and misses the direct molecular players. However, proximity labeling cuts directly to the heart of the interaction by biotinylating and identifying the host proteins that physically neighbor the bacterial surface or its secreted effector proteins. This reveals the initial interaction interface itself. Bioinformatic predictions are useful for generating hypotheses, but they only provide computational insights whose mechanistic strength and reliability require experimental validation. Proximity labeling is a powerful, high-throughput validation platform. By expressing the labeling enzyme in a pathogen of interest, researchers can empirically discover and validate the host pathways being manipulated, transforming a list of candidate interactors into a molecular map with confidence. Thus, by enabling the direct, quantitative capture of protein-protein interactions *in situ* within living systems, proximity labeling provides an unparalleled advantage in transitioning from the observation of correlations to the definition of causal mechanisms at the microbe-host interface.

**Figure 4:**
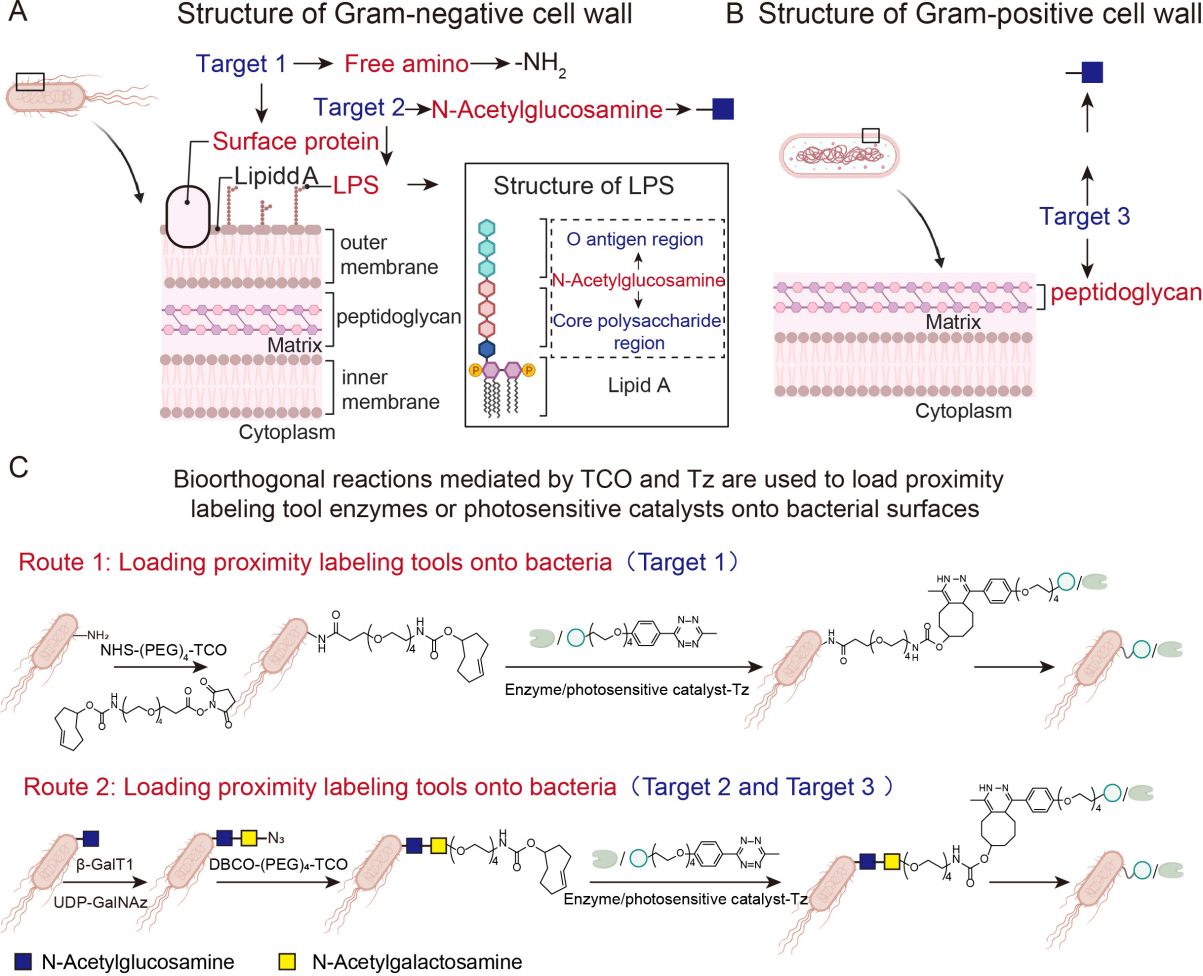
**(A, B)** Schematic diagram of cell wall structures of Gram-negative and Gram-positive. **(C)** Schematic diagram showing how a proximity labeling enzyme or photocatalyst is loaded onto the bacterial surface.

The first step in investigating microbe-host cell interactions within the tumor microenvironment using proximity labeling technology is to load the proximity labeling tool enzyme or photosensitized catalyst onto the bacterial surface. Model bacteria are usually susceptible to genetic editing, enabling the engineered expression of tool enzymes on their surfaces. However, primary pathogenic bacteria such as *Helicobacter pylori* and *Staphylococcus aureus* generally present significant challenges to efficient genetic editing. Although metabolic labeling can also load tool enzymes or photocatalysts onto bacterial surfaces, this approach requires the bacteria to be exposed to media containing high concentrations of labeling agents. This disrupts bacterial metabolic processes and affects their intrinsic interactions with host cancer cells. Consequently, this method is not suitable for modifying bacteria in order to study their interactions with host cells. Therefore, a method that can efficiently and broadly load proximity labeling tool enzymes or photocatalysts onto bacterial surfaces must be developed. Considering the different primary components of Gram-positive and Gram-negative bacterial cell walls: Gram-positive cell walls have a simple structure consisting primarily of peptidoglycan, which is made up of polysaccharide and cross-linked polypeptide chains. In contrast, Gram-negative cell walls have a complex composition consisting of an inner layer of peptidoglycan and an outer layer of lipids, lipopolysaccharides and proteins. Lipopolysaccharides comprise lipopolysaccharide A, core polysaccharides and O antigens. Core polysaccharides are a conserved region, whereas O antigens typically exhibit greater variation. Based on these structural characteristics, the free amino groups on the surface of Gram-negative bacterial cell walls can be selected as loading targets for proximity-tagging tool enzymes or photosensitizing catalysts Target 1. N-acetylglucosamine, which is found in both the O-antigen and core polysaccharide regions of the polysaccharide, can serve as loading target 2. For Gram-positive bacteria, free N-acetylglucosamine on the peptidoglycan can be selected as loading target 3 (**Figures 4A, B**). For target 1, trans-cyclooctene (TCO) groups were modified onto the amines of the bacterial surface using trans-cyclooctene-tetra-PEG-hydroxysuccinimide ester (NHS-(PEG)₄-TCO). For targets 2 and 3, catalyzed by β-galactosyltransferase (β-GalT1), N-azidoacetyl-N-acetylglucosamine (GalNAz) tetracarboxylated with guanosine diphosphate (UDP-GalNAz) reacted with N-acetylglucosamine on the bacterial surface to modify azido (N₃) groups onto the bacterial surface. Subsequently, dibenzocyclooctyne-polyethylene glycol-trans-cyclooctene (DBCO-(PEG)₄-TCO) reacted with the N₃ groups, modifying the TCO groups onto the bacterial cell wall. These chemically modified tools enable bioorthogonal Tz groups to be loaded onto tool enzymes or photosensitive catalysts. Bacteria bearing the TCO group then react with nearby labeled tool enzymes or photosensitizers carrying Tz groups. This bioorthogonal reaction enables tool enzymes or photosensitizers to be loaded onto the bacterial surface (**Figure 4C**). This design facilitates the loading of nearby labeled tool enzymes or photocatalysts onto both Gram-negative and Gram-positive bacteria.

Proximity labeling technology enables the detection of interactions with universality, quantitative capability, high selectivity and high resolution. Advantages of using this technology to study microbe and host cell interactions include high temporal and spatial resolution, strong environmental compatibility and covalent labeling to generate interaction signals that are difficult to lose and compatible with multiple omics (Deng et al.; Nakandakari-Higa et al., 2024). These key advantages make proximity labeling technology a powerful tool for studying microbe-host cell interactions within the tumor microenvironment.

## Opportunities and challenges

### Opportunities

**Figure 5:**
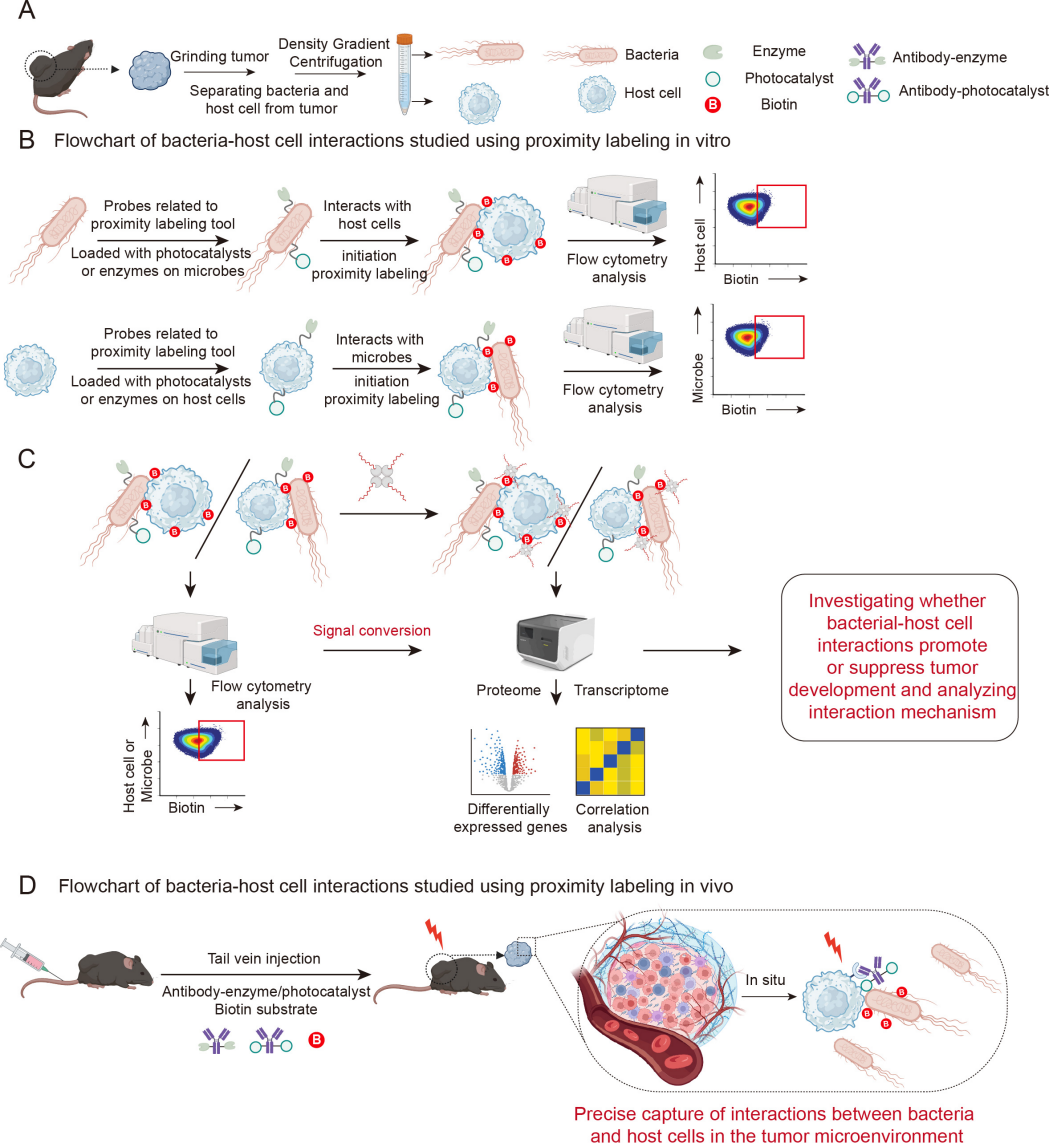
Schematic diagram showing microbe-host cell interactions in the tumor microenvironment using proximity labeling technology. **(A)** Schematic diagram showing the separation of bacteria and host cells from tumor. **(B)** Schematic illustration of two approaches to capturing bacterial-host cell interactions *in vitro* using proximity labeling technology. **(C)** Schematic diagram illustrating the conversion of biotin signals representing interaction strength into DNA signals suitable for sequencing. **(D)** Schematic illustration of capturing bacterial-host cell interactions *in vivo* using proximity labeling technology.

Proximity labeling tools can be used to study the interactions between microbe and host cancer or immune cells within the tumor immune microenvironment. When enzymes or photocatalysts are loaded onto bacterial surfaces, the interactions and mechanisms between the bacteria and host cells can be studied *in vitro*. Bacteria and host cells within tumors can be separated via density gradient centrifugation (**Figure 5A**). Chemically modified strategies are employed to load tool enzymes or photocatalysts onto bacterial surfaces (**Figure 5B**). Interaction with host cells *in vitro* triggers proximity labeling reactions by detecting biotin labeling on different host cell types, providing information on bacterial-host cancer cell interactions. Bioinformatics approaches such as average transcriptome sequencing or single-cell transcriptomics can then be employed. Proximity labeling techniques can generate biotin signals on host cells, representing the strength of interactions between microorganisms and host cells. The biotin-streptavidin interaction is the strongest and most specific covalent bond in biotechnology. Using streptavidin-modified single-stranded DNA conjugates (SA-ssDNA) converts the biotin signal reflecting the strength of microbial-host cell interaction into a sequence-readable ssDNA signal. At the single-cell level, this approach analyses the strength of bacterial-host cancer cell interactions and gene or protein expression level changes across different cells (**Figure 5C**). It maps altered states in various host cells, including cancer and immune cells, following bacterial engagement. This process discerns whether these interactions inhibit or promote tumorigenesis, and elucidates the underlying mechanisms that govern their impact on tumor progression.

Of course, enzymes or photocatalysts can also be loaded onto host cells (**Figure 5B**). Compared to loading tools onto bacteria, this approach offers a wider selection of targets and more diverse methods. This enables the simultaneous study of interactions between bacteria and host cells *in vivo* and *in vitro*. The process of studying bacterial-host cell interactions *in vitro* by loading tools onto cells is consistent with the process for bacteria. Both require tumor tissue extraction, followed by density gradient centrifugation to separate the bacteria from the host cells. However, host cell membranes offer more modifiable targets, such as proteins, phospholipids and glycoproteins. Molecules that target these sites can be conjugated with tool enzymes or photosensitizers in order to attach the tool to specific host cell targets. Subsequent proximity labeling reactions capture interactions between host cells and bacteria. This approach can identify novel bacteria that interact with host cancer cells. Downstream integration with single-bacterial transcriptome analysis can elucidate whether these interactions promote or suppress tumorigenesis, thereby revealing the underlying mechanisms. Furthermore, loading tools onto the host cell surface offers the significant research advantage of enabling the precise *in situ* capture of bacterial-host cell interactions within tumors. For example, in the mouse triple-negative breast cancer model E0771, near-infrared proximity labeling tools such as Sn^IV^ catalysts (Buksh et al., 2022), pyro pheophorbide (Deng et al.) or PCN nanozymes (Li et al., 2025) can be used as *in vivo* research tools. These can be conjugated with the anti-Her2 antibody, Herceptin, and the biotin-substrate probe conjugate can be directly injected into tumor-bearing mice and exposed to infrared light ex vivo (**Figure 5D**). This approach enables the direct *in vivo* capture of bacteria or other microbes interacting with Her2-positive E0771 cells within the tumor microenvironment. In addition to loading tool enzymes or photocatalysts into cancer cells, this strategy can be applied to any cell type within the tumor microenvironment, such as T cells, B cells, NK cells, macrophages and dendritic cells, to identify the specific bacteria interacting with target cells and to uncover their critical roles in tumor progression. This research approach has the potential to discover specific bacteria *in situ* within the tumor microenvironment and explore their novel functions.

**Figure 6:**
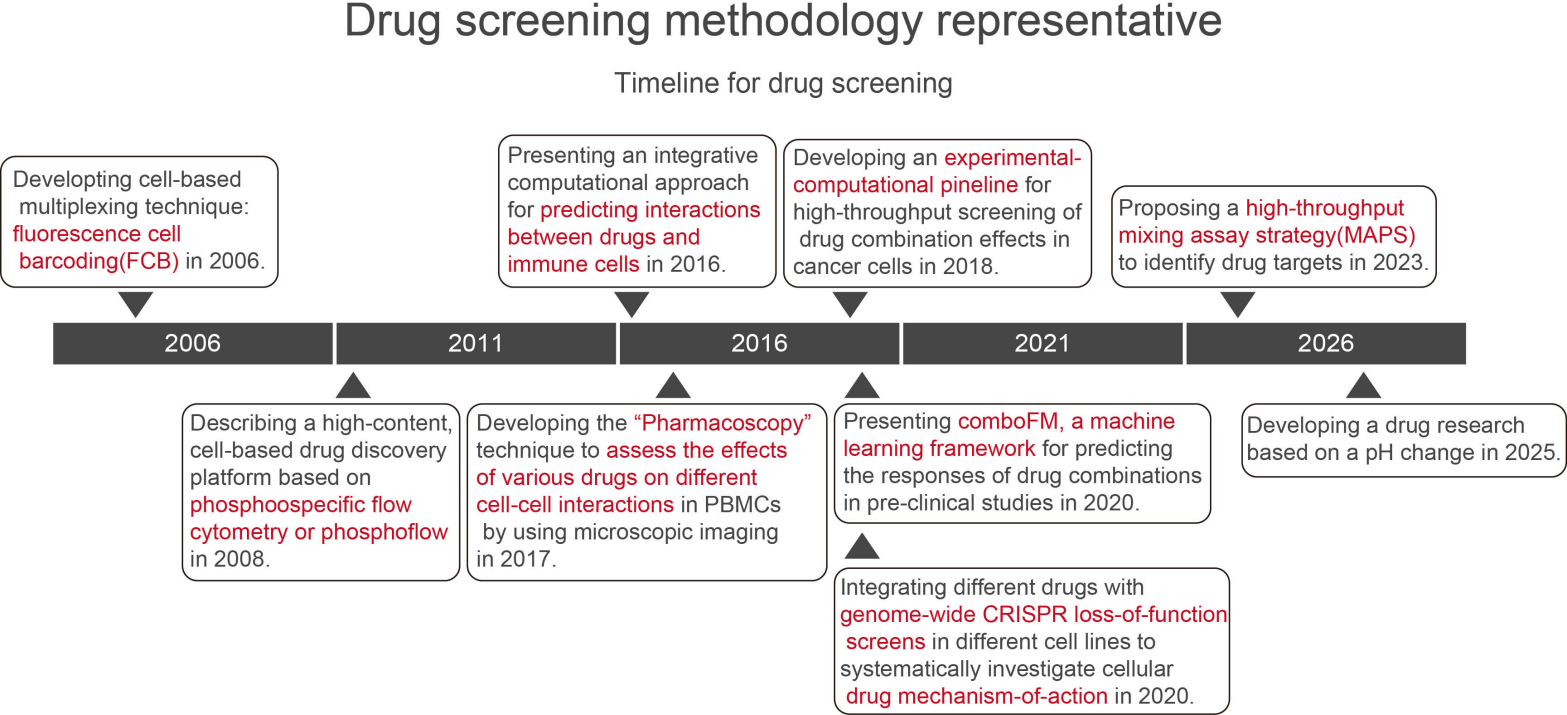
Current approaches to drug screening research.

Proximity labeling technique can be used to identify interactions between bacteria and host cancer cells within the tumor microenvironment. This technique can also be used to quantitatively characterize cell-cell interactions, we can use proximity labeling to quantitatively assess these interactions during the capture of bacterial-host cancer cell interactions. Once it is understood whether these interactions promote or inhibit tumorigenesis, progression and metastasis, the quantitative nature of this technology can be leveraged to screen drugs that modulate them. Based on the strength of the interaction and its impact on tumor development, drugs can be selected to either enhance or diminish these interactions. If bacterial-host interactions promote tumorigenesis, drugs that weaken these interactions should be selected. Conversely, if they inhibit tumorigenesis, drugs that enhance these interactions should be chosen. Bioinformatics analysis can then be used to elucidate the mechanisms by which drugs modulate these interactions. Screening drugs using proximity labeling technology has several advantages compared to existing research approaches, such as: fluorescence cell barcoding (FCB) for high-throughput drug screening and signaling profiling (Krutzik and Nolan, 2006); cell-based high-throughput drug discovery platforms (Krutzik et al., 2008); integrated computational approaches for predicting drug-immune cell interactions (Kidd et al., 2016); high-throughput microscopic imaging technology termed “Pharmacoscopy” (Vladimer et al., 2017); comboFM strategies (Julkunen et al., 2020); genome-wide CRISPR loss-of-function screening for drug mechanisms (Gonçalves et al., 2020); high-throughput mixed assay strategies (Ji et al., 2023); pH-based drug screening methods (Cordeiro et al., 2025) (**Figure 6**). The methodology’s key strength lies in its ability to quantitatively capture bacteria-host cancer cell interactions in real time and elucidate their mechanisms.

Consider, for instance, the specific “intracellular bacteria” identified by Cai et al. in naturally occurring breast tumors in mice, which promote tumor metastasis within breast tissue (Fu et al., 2022). If drugs that block the interaction between intracellular bacteria and host cancer cells could be screened *in vitro* using proximity labeling technology at the moment of interaction, bacterial invasion of host cancer cells could be prevented, significantly suppressing tumor metastasis. This would provide a universal and efficient approach to advancing drug discovery research aimed at preventing tumor progression and metastasis. Furthermore, photocatalytic proximity labeling techniques typically generate singlet oxygen during application. Combining these with proximity-labeled lethal strategies to promote the death of cancer-promoting bacteria offers a cutting-edge approach to inhibiting tumor progression and metastasis.

Susan Bullman’s research team at the University of Texas, for instance, revealed that tumor-infiltrating bacteria disrupt interactions between rectal or oral cancer epithelial cells and induce cell cycle arrest, thereby affecting the prognostic response to chemotherapy (Galeano Niño et al., 2025). Screening drugs capable of weakening interactions between tumor-infiltrating bacteria and epithelial cells using proximity labeling strategies *in vitro* and administering them prior to chemotherapy to block bacterial disruption of epithelial cells could improve the prognostic response to chemotherapy. This represents an important research direction for enhancing the efficacy of chemotherapy.

### Challenges

Although proximity labeling technology offers multifaceted research opportunities for studying bacterial-host cell interactions within the tumor immune microenvironment, it also presents a series of challenges. For example, given the different cell wall structures of Gram-positive and Gram-negative bacteria, we have currently identified only three loadable targets and two loading strategies. This contrasts with the multitude of loadable targets present on cells, thereby limiting the achievable bacterial modification methods with proximity labeling tools. Therefore, when studying bacterial-host cell interactions in the tumor immune microenvironment, the tumor must first be excised, ground and separated to isolate bacteria from host cells before bacterial modification if tool enzymes or photocatalysts are loaded onto bacterial surfaces. This process requires the re-establishment of interactions between the two entities, which prevents direct *in situ* capture of their interactions within the tumor. Consequently, critical *in situ* interaction information may be overlooked. Current methods can only modify tumor cells with tool enzymes or photosensitizers to capture bacterial-host interactions *in situ* within the tumor immune microenvironment using proximity labeling. As described above in the E0771 tumor model, the Her2 protein on the tumor cell surface was selected as the loading target for the tool. Using the targeting function of antibodies, the tool enzyme or photosensitizing catalyst was loaded onto the cancer cell surface, thereby enabling the capture of interactions *in situ* between bacteria and host cells within the tumor site. Future research employing proximity labeling techniques to study bacterial-host interactions within tumors should explore additional bacterial loading targets. If proximity labeling tools could be loaded onto bacterial surfaces *in situ* within the tumor, the captured interactions would be more precise, reducing the risk of overlooking them.

## Summary and outlook

Proximity labeling technologies are characterized by the universality, quantitative, high selectivity and high resolution of their interaction detection. Therefore, proximity labeling technology has the potential to be a valuable tool for quantitatively studying microbe-host cell interactions in the tumor microenvironment. Due to its unique properties, this technology can be used to decipher the regulatory mechanisms by which drugs influence the interactions between microbes and host cells within the tumor immune microenvironment. Its operational workflow enables the precise capture of dynamic changes in these interactions during drug intervention. The effects of drugs such as immunomodulators, receptor antagonists, enzyme inhibitors or antibiotics on the interactions between microbes and host cancer cells within the immune microenvironment are highly complex. Differences in the timing and stage at which drugs are applied to bacteria or host cells can lead to markedly distinct interaction outcomes. Evaluating the effects of drugs at a single point in the interaction pathway risks overlooking critical information.

**Figure 7:**
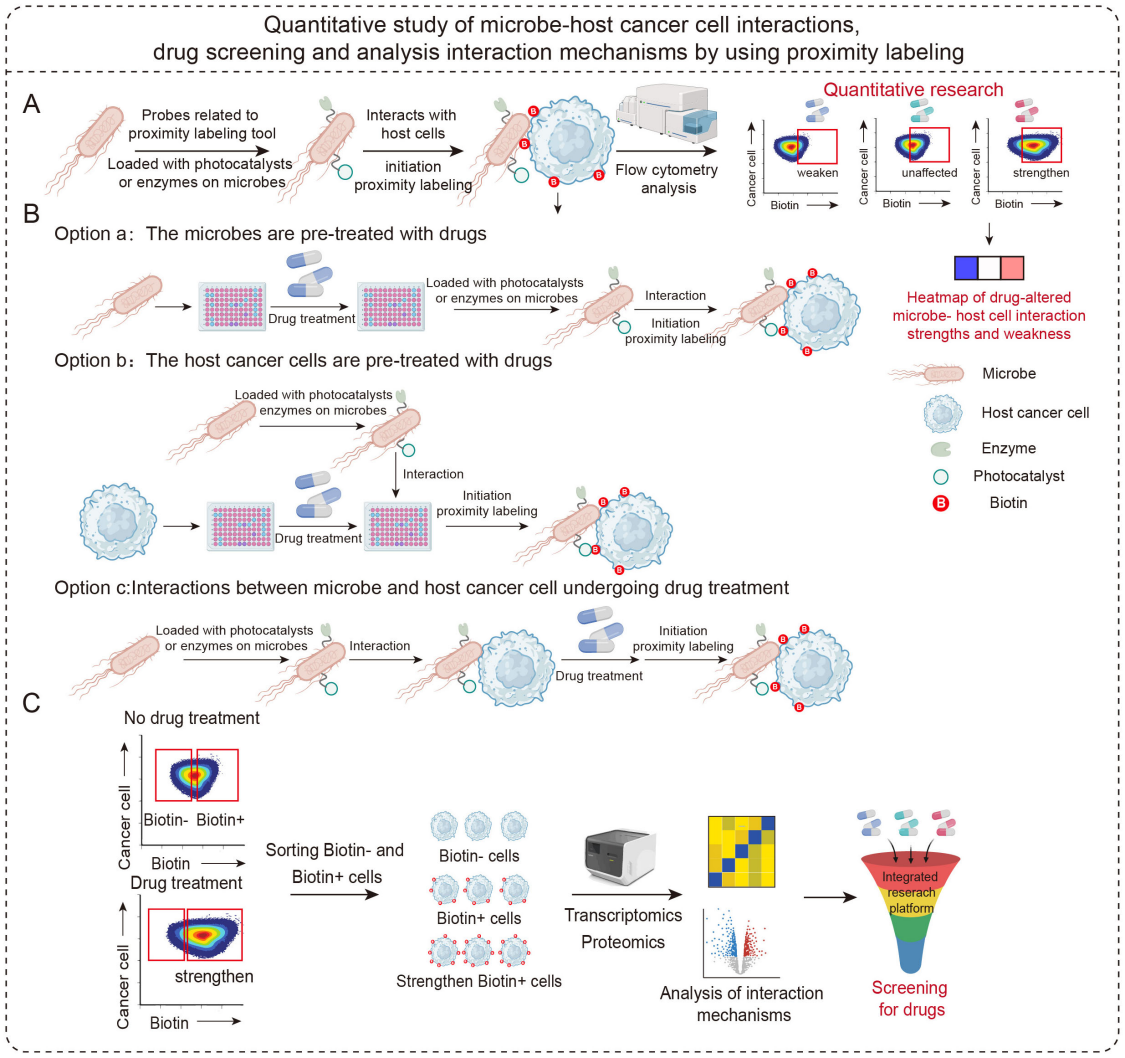
Schematic diagram showing the proximity labeling to study interactions between microbes and host cells, and then to screen drugs and mechanisms affecting these interactions. **(A)** Scheme of the quantitative study of microbe-host cell interactions using proximity labeling technology. **(B)** Scheme of drug screening utilizing proximity labeling technology to identify drugs affecting microbe-host cell interactions. **(C)** Scheme of microbe-host cell interactions and mechanisms of change analyzed by combining proximity labeling technology with multi-omics technologies.

Researchers can use proximity labeling technology to conduct a systematic and comprehensive investigation of the impact of drugs on microbial-host cancer cell interactions (**Figure 7A**). Specifically, three experimental approaches enable in-depth analysis: First, pre-treating microbes with drugs before co-culturing them with host cells carrying proximity labeling tools captures drug effects on interactions via labeling reactions. Second, pre-treating host cancer cells with drugs before introducing microbes carried proximity labeling tools analyses interaction changes under drug regulation. Third, equipping microbes with proximity labeling tools before inducing interactions with host cells captures the interaction process in real time under drug exposure. Combining these three approaches enables the effective differentiation of primary drug targets (microbes, host cells, or both), providing a powerful tool for elucidating the mechanisms by which drugs regulate microbe-host cancer cell interactions (**Figure 7B**). At the same time, this technology can easily and rapidly convert abstract microbe-host cell interactions into visual biotin signals and be easily combined with downstream bioinformatics tools. Understanding the mechanism of microbe-host cell interaction is helpful for understanding how such interactions can be intervened in and regulated by different types of drug molecules. Advantages of using proximity labeling technology to study microbe-host cell interactions and mechanisms include high temporal and spatial resolution, strong environmental compatibility, and covalent labeling to generate interaction signals that are difficult to lose and compatible with multiple omics (**Figure 7C**). Improving researchers’ ability to identify and manipulate these interactions in the tumor microenvironment will provide vital insights into tumor immunotherapy and human health, ultimately accelerating the development of this therapeutic approach.
